# Major complications of sigmoid vaginoplasty: a case series

**DOI:** 10.1093/jscr/rjad333

**Published:** 2023-06-13

**Authors:** Matthew S Meece, Lee E Weber, Alexandra E Hernandez, Sara J Danker, Nivedh V Paluvoi

**Affiliations:** DeWitt Daughtry Family Department of Surgery, Jackson Memorial Health System, Miami, FL, USA; Department of Surgery, Division of Colorectal Surgery, University of Miami, Miami, FL, USA; DeWitt Daughtry Family Department of Surgery, Jackson Memorial Health System, Miami, FL, USA; Department of Surgery, Division of Plastic Surgery, University of Miami, Miami, FL, USA; DeWitt Daughtry Family Department of Surgery, Jackson Memorial Health System, Miami, FL, USA; Department of Surgery, Division of Colorectal Surgery, University of Miami, Miami, FL, USA; DeWitt Daughtry Family Department of Surgery, Jackson Memorial Health System, Miami, FL, USA; Department of Surgery, Division of Plastic Surgery, University of Miami, Miami, FL, USA; DeWitt Daughtry Family Department of Surgery, Jackson Memorial Health System, Miami, FL, USA; Department of Surgery, Division of Colorectal Surgery, University of Miami, Miami, FL, USA

## Abstract

This case series explores the major complications following sigmoid vaginoplasty in two transgender female patients. Both patients experienced significant post-operative complications, including stenosis and abscess formation, leading to sigmoid conduit ischemia and necrosis. These complications required major surgical interventions and multidisciplinary care, highlighting the complexity of these procedures and their potential morbidity. Our analysis suggests that the initial stenotic insult led to obstruction and vascular insult to the sigmoid conduit, necessitating resection of the affected bowel. The outcomes underscore the need for collaboration across specialties for optimal post-operative monitoring and management. This study advocates for future management guidelines promoting multidisciplinary collaboration to reduce morbidity and resource burdens associated with complications. Despite the complications, sigmoid vaginoplasty remains a viable gender affirmation surgery, providing an effective analogue to vaginal mucosa and offering improved neovaginal depth.

## INTRODUCTION

The accessibility of experienced providers offering gender affirmation surgery is ever expanding and is an important part in the transition process of transgender patients. Currently, affirmation surgery for transgender females is achieved with either of two procedures: penile inversion or sigmoid vaginoplasty [1]. The modality patients and physicians choose depends on multiple factors including, but not limited to surgical invasiveness, penile length, introitus depth, appearance, and lining [2]. Regardless of technique choice, the authors believe that for optimal results all cases should be done with close multidisciplinary collaboration, in both the intraoperative and post-operative period. Herein, we present a series of two cases where such a collaboration between specialties proved important in the ultimate care of patients and advocates for future management guidelines to do the same.

## CASE SERIES

### Patient A

Patient A underwent primary sigmoid vaginoplasty in 2017. Her recovery was complicated with cellulitis and prolonged urinary retention on post-operative Days 19 and 20, respectively, which were managed with irrigation and drain placement, along with transposition of the neo-urethra per urological surgery. Following this, the patient had no issues and continued with dilation and clinic follow-up. One year post-operatively, she presented to the emergency department with abdominal pain, vomiting, fever, leukocytosis, large volume mucinous discharge, and an inability to dilate. CT imaging found abrupt narrowing of the rectosigmoid junction with a large fluid collection, with additional loculated collections in the pelvis and mid-abdomen concerning for abscesses. There immediately was concern for micro-perforation or anastomotic breakdown at the sigmoid conduit, and intravenous antibiotics were started, along with close monitoring by colorectal and plastic surgery. An exam under anaesthesia found a significant vaginal stricture that was subsequently dilated and an endoscope was inserted. Immediately, the scope identified a new cavity proximal to the stricture, which was surrounded by fat and muscle rather than expected colonic mucosa, and the decision for further exploration with colorectal surgery was made. Midline laparotomy revealed a 2 cm perforation of the sigmoid conduit which appeared necrotic, in addition to multiple dense adhesions of the small bowel and right colon in the pelvis. This necrotic section was resected at the level of the staple line to the phallus skin, and subsequently removed. Intraoperatively, multiple interloop abscesses were present and the patient required an extended course of intravenous antibiotics during her stay and recovery. Eventually, she was stabilized and discharged and began dilation at her third week post-operatively and continued to dilate without further complication.

### Patient B

**Figure 1:**
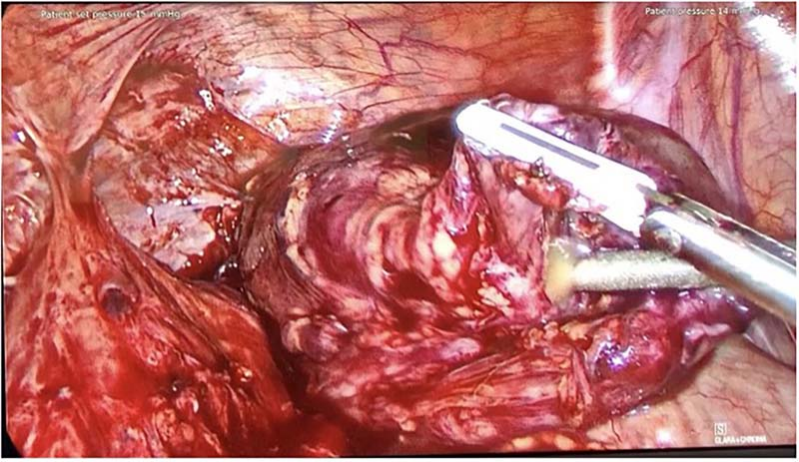
Intraoperative photo from Patient B, showing conduit perforation with purulent exudate.

**Figure 2:**
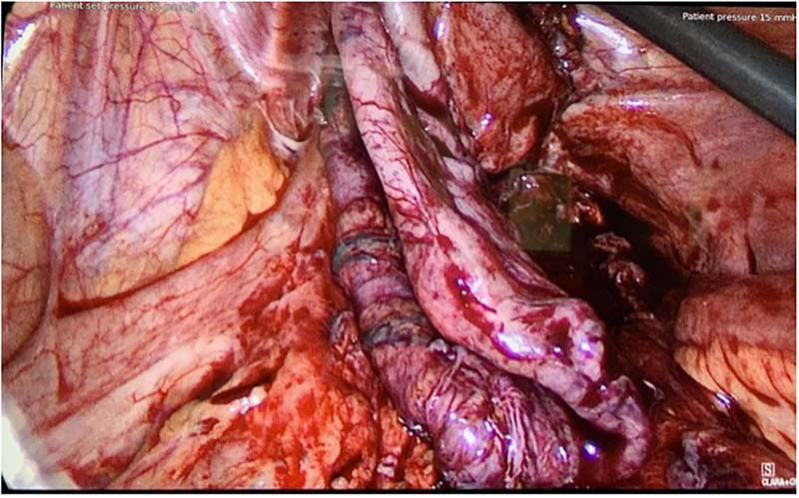
Intraoperative photo from Patient B, showing a segment of ischemic sigmoid conduit.

Patient B underwent primary sigmoid vaginoplasty in 2016. One month post-operatively, she was found to have vaginal stenosis secondary to a high riding perineum, which was repaired with a midline incision and internal suturing towards the colon flap. Following this, the patient recovered and resumed dilation without complication. She presented 3 years later with abdominal pain, fever, nausea, vomiting and leukocytosis. CT imaging demonstrated a dilated sigmoid conduit. Exam under anaesthesia revealed a completely occluded neovagina at the phallo-collic anastomosis. Confirmation by laparoscopy revealed the sigmoid conduit to be severely dilated, ischemic, with dense small bowel adhesions at the proximal portion. There was also evidence of perforation at the most proximal aspect of the conduit (Figs 1 and 2). The decision was made to resect the sigmoid conduit at this point. Her subsequent recovery was uncomplicated, and the patient resumed dilation 3 weeks post-operatively, continued without issue until her follow-up became as needed.

## DISCUSSION

Sigmoid vaginoplasty, also referred to as rectosigmoid neocolporrhaphy or neocolpopoesis, was first described for use in transgender patients by Markland and Hastings [3]. Since then, it has become an increasingly popular technique for gender affirmation surgery [2]. Currently, the surgical technique has been described in both and open and laparoscopic fashion [2]. In many institutions, including our own, employing colorectal surgeons to identify, harvest and assist in insetting of an adequately perfused sigmoid segment is a crucial portion of this procedure [4]. The invasive nature of sigmoid vaginoplasty, including its intraabdominal and pelvic portions leads to numerous risks of surgical complications. In the vast majority of patients, these complications are minimal and are handled with minor clinical changes or surgical procedures [4–8]. A recent large meta-analysis identified the individual complication rates, including fistula (2%), stenosis/strictures (14%), tissue necrosis (1%) and prolapse (4%) with overall satisfaction rate of 93% [8]. Colonic vaginoplasty and its related stenosis of the neovagina are divided into two types: introital stenosis and diffuse stenosis. A review found no correlation between occurrence of stenosis and type of incision used, use of introital flaps, dilation protocol, or sexual activity [7]. Both cases we describe presented with diffuse stenosis with unknown initial insult, complicated by ischemia in the sigmoid conduit requiring subsequent resection of affected bowel. We presume that the initial stenotic insult of the introitus in both cases led to the sigmoid conduit to become obstructed with subsequent vascular insult and perforation. The interventions in these patients were a combined plastic and colorectal surgical approach. While curative, these patients suffered further cosmetic and functional morbidity, namely, a reduced depth of neovagina with loss of the colonic segment, surgical access scarring and post-operative infection.

For gender affirming surgery for transfemales, use of the sigmoid colon provides a good physiologic analogue to vaginal mucosa while also increasing depth of the neovagina in comparison to other procedures. With these benefits come increased surgical complexity, increased risk of post-operative complication and requirement for collaboration amongst multiple subspecialties to offer optimal functional and cosmetic results with minimal patient morbidity. It is imperative that this dual treatment modality be maintained to monitor patients post-operatively for any complication that may arise. We believe that in doing so, we can offer the patient the best result and reduce the resources and burden patients may undertake if complications may arise.

## CONFLICT OF INTEREST STATEMENT

None declared.

## FUNDING

None.
